# Genome-Wide Transcriptional Excavation of *Dipsacus asperoides* Unmasked both Cryptic Asperosaponin Biosynthetic Genes and SSR Markers

**DOI:** 10.3389/fpls.2016.00339

**Published:** 2016-03-29

**Authors:** Jian-ying Wang, Yan-li Liang, Mei-rong Hai, Jun-wen Chen, Zheng-jie Gao, Qian-qian Hu, Guang-hui Zhang, Sheng-chao Yang

**Affiliations:** Yunnan Research Center on Good Agricultural Practice for Dominant Chinese Medicinal Materials, Yunnan Agricultural UniversityYunnan, China

**Keywords:** *Dipsacus asperoides*, Illumina, CYP450s, UGTs, SSRs

## Abstract

**Background:** *Dipsacus asperoides* is a traditional Chinese medicinal crop. The root is generally used as a medicine and is frequently prescribed by Chinese doctors for the treatment of back pain, limb paralysis, flutter trauma, tendon injuries, and fractures. With the rapid development of bioinformatics, research has been focused on this species at the gene or molecular level. For purpose of fleshing out genome information about *D. asperoides*, in this paper we conducted transcriptome analysis of this species.

**Principal Findings:** To date, many genes encoding enzymes involved in the biosynthesis of triterpenoid saponins in *D*.asperoides have not been elucidated. Illumina paired-end sequencing was employed to probe *D. asperoides*'s various enzymes associated with the relevant mesostate. A total of 30, 832,805 clean reads and *de novo* spliced 43,243 unigenes were obtained. Of all unigenes, only 8.27% (3578) were successfully annotated in total of seven public databases: Nr, Nt, Swiss-Prot, GO, KOG, KEGG, and Pfam, which might be attributed to the poor studies on *D. asperoides*. The candidate genes encoding enzymes involved in triterpenoid saponin biosynthesis were identified and experimentally verified by reverse transcription qPCR, encompassing nine cytochrome P450s and 17 UDP-glucosyltransferases. Specifically, unearthly putative genes involved in the glycosylation of hederagenin were acquired. Simultaneously, 4490 SSRs from 43,243 examined sequences were determined via bioinformatics analysis.

**Conclusion:** This study represents the first report on the use of the Illumina sequence platform on this crop at the transcriptome level. Our findings of candidate genes encoding enzymes involved in Dipsacus saponin VI biosynthes is provide novel information in efforts to further understand the triterpenoid metabolic pathway on this species. The initial genetics resources in this study will contribute significantly to the genetic breeding program of *D. asperoides*, and are beneficial for clinical diagnosis and treatment.

## Introduction

*Dipsacus asperoides* is a traditional Chinese medicinal crop of the Dipsacaceae family. Its root (Supplementary File S1) gets its name “Dipsaci radix” from the capacity to heal broken bone, and is considered “top grade” as early as Shen Nong's *Herbal Classic*. More detailed, related information can be found in the *Pharmacopoeia of the People's Republic of China* (Beijing, 2010, Vol I, p. 309). Later generations often use it as a prescription with other compatible medicines. It has been used clinically for the treatment of osteoporosis, lassitude in loin and legs, fractures, abortion and dysmenorrheal diseases, as well as Alzheimer's disease and cancer (Zhang et al., 1997, 2003; Corcelle et al., 2006; Wong et al., 2007; Zhu et al., 2009; Seifert-Klauss and Prior, 2010; Niu et al., 2015). Thus, it is a promising medicinal plant.

Over recent decades, the demand for *D. asperoides* has continued to rise. *D. asperoides* is a perennial herb (Supplementary File S2), and there is an increasing disparity between its long growth cycle and excessive harvesting of wild populations. To alleviate this conflict, on the one hand, it is imminent to focus on breeding improved varieties at molecular level in order to respond to a variety of coercive influences; on the other hand, it is also urgently needed to produce the bioactive ingredient via genetic engineering to meet the ever-growing demand for this herbal alternative.

Currently, although next generation sequencing (NGS; Suter et al., 2015) has been very broadly applied to RNA-Seq in a large number of plant species, *de novo* (Moreton et al., 2015) transcriptome sequencing still has not utilized the reference approach to excavate sufficient useful genomic information (Grabherr et al., 2011). This is due to the absence of reference genomes for non-model plants. In this study, we made some improvements by assembling together all the clean reads into a transcriptome and used as reference sequences in the follow-up analysis. As expected, RNA-Seq results gave many clues concerning genetic and molecular marker information for *D. asperoides*. As the first report on whole genome sequencing of *D. asperoides*, the sequencing results will significantly help the analysis of a genome-wide study in this species.

Although it has been over thousand years since *D. asperoides* was used as an important common traditional Chinese medicine, its various active ingredients have still not been elucidated. In Europe, North Africa and Asia, *D. asperoides* is one kind of widely distributed herb. In China, Yunnan, Sichuan, Hubei and Hunan Provinces are the main origins of *D. asperoides*. Existing studies show that *D. asperoides* has a variety of pharmacological activities, which are primarily attributed to saponin compounds, the active components in root of *D. asperoides*. Triterpenoid saponins (Supplementary File S3) (Liu et al., 2011) such as HN saponin F, Dipsacus saponin VI and Macranthoidin A are the principal active ingredients in this plant. Akebiasaponin D (i.e., Dipsacus saponin VI) is an important quality indicator of *D. asperoides* (Liu et al., 2010). However, the metabolic pathways of this compound are still unknown (even though extensive research on this compound has been reported).

**Figure 1:**
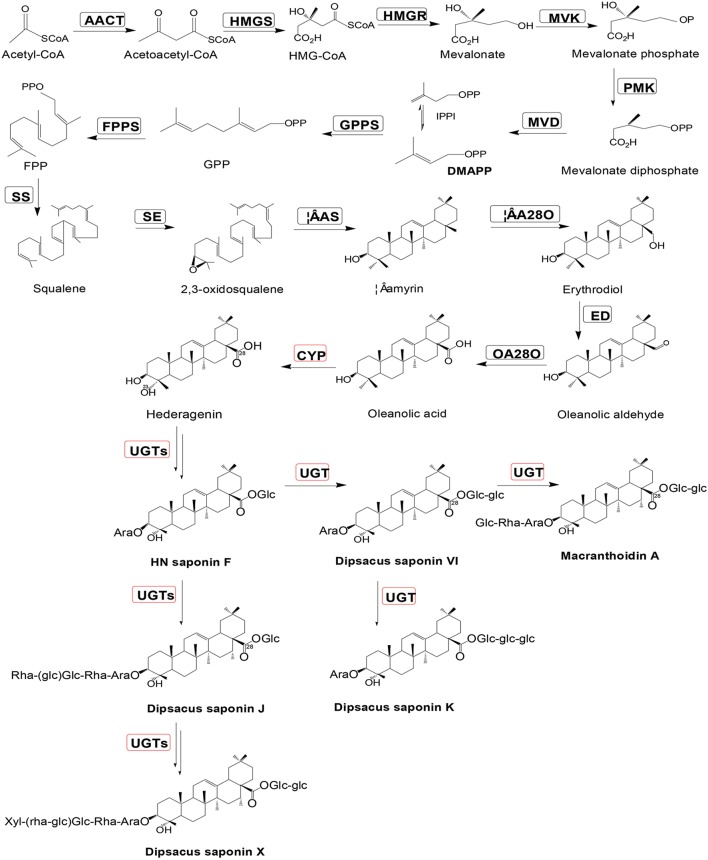
**Putative Dipsacus saponin VI biosynthesis pathway in *D. asperoides***. Enzymes found in earlier studies are surrounded by a black box; new putative enzymes are surrounded by a red box. Enzymes involved in the pathways are: AACT, acetyl-CoA acetyltransferase; HMGS, hydroxymethylglutaryl-CoA synthase; HMGR, hydroxyl methylglutaryl-CoA reductase; MVK, mevalonate kinase; PMK, phosphomevalonate kinase; MVD, mevalonate diphosphate decarboxylase; GPPS, geranylgeranyl pyrophosphate synthase; FPPS, farnesyl diphosphate synthase; IPPI, isopentenyl diphosphate isomerase; SS, squalene synthase; SE, squalene epoxidase; β-AS, β-amyrin synthase; and β-A28O, β-amyrin 28-monooxigenase.

The purpose of this study was to examine the transcriptome of *D. asperoides* using Illumina second-generation sequencing platforms (Fu et al., 2013), as well as to mine all genes encoding enzymes involved in biosynthetic pathways of Dipsacus saponin VI. The proposed synthetic routes were shown in Figure 1. NGS (Strickler et al., 2012) technologies allow us to dissect the entire transcriptome of specific species without model plants (Grabherr et al., 2011), and subsequently enable us to access information about biological pathways and disease mechanisms. This information also included gene function, single nucleotide polymorphisms (SNP; Somers et al., 2003) calling, Simple Sequence Repeat (SSR; Ramsay et al., 2000) markers of one species, and so on. The present study will contribute to the improvement of genetic diversity in germplasm resources of *D. asperoides* and the pharmacological biosynthesis of the active components of this plant *in vitro*.

## Results and discussion

### Reads acquirement and *de novo* assembly

Based on cDNA library construction, Illumina Genome Analyzer IIx produced 30,832,805 clean reads with 97.28% of Q20 percentage (Cock et al., 2010). Thus, of all clean reads, the proportion with 99% correctly identification accounted for 97.28%—an ideal sequencing result. All sequencing reads were entered in the NCBI web site and could be accessed with the short read archive number of SRA269859. Using Trinity software (Grabherr et al., 2011), the clean reads were then assembled into 73,036 contigs (Seong et al., 2015) with total length of 59,560,527 bp. The lengths of all contigs covered a range of 201–7591 bp, with a mean length of 815 bp and a N50 size (Earl et al., 2011) of 1262 bp. All of the above contigs were assembled into 43,243 unigenes with a total length of 31,420,741 bp. The range of the lengths of all genes was similar to the contigs, with a mean length of 727 bp and a N50 size of 1212 bp. All relevant Illumina paired-end sequencing and assembly data are summarized in Table 1.

**Table 1:** **Summary of Illumina paired-end sequencing and assembly for *D. asperoides***.

	**Total number**	**Total length (bp)**	**Min length (bp)**	**Mean length (bp)**	**Max length (bp)**	**N50 (bp)**	**Total clean reads (bp)**	**Q20 (%)**	**GC (%)**
Contig	73,036	59,560,527	201	815	7591	1262	–	–	–
Unigene	43,243	31,420,741	201	727	7591	1212	30,832,805	97.28	43.94

**Figure 2:**
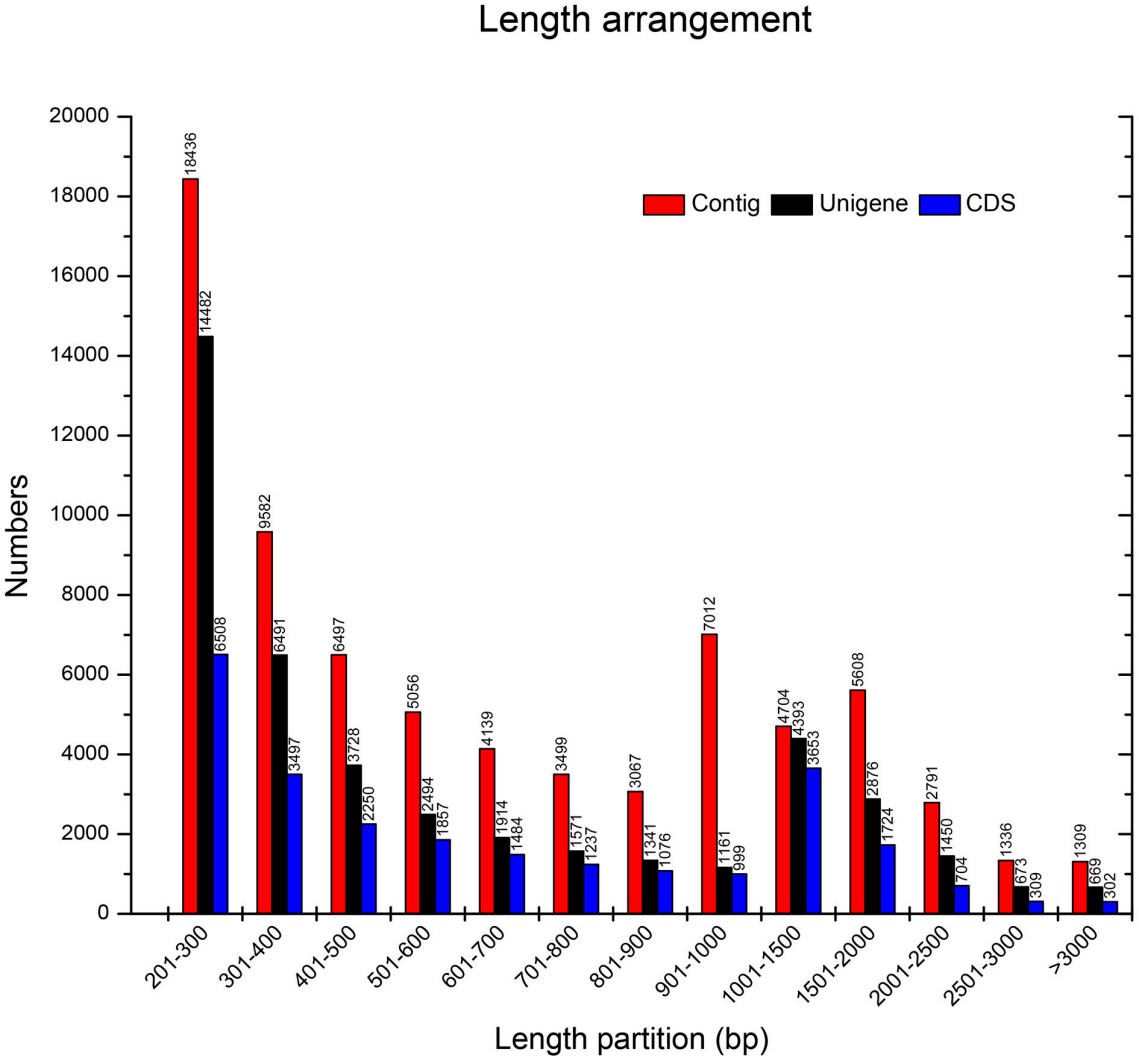
**Overview of the *D. asperoides* transcriptome assembly and the length distribution of the Contigs, Unigenes, and Coding sequences (CDS)**.

All unigenes were compared with the whole set of public protein databases using BLASTX (*E*-value from 1e–10 to 1e–3), i.e., Nr (NCBI non-redundant protein sequences), Nt (NCBI non-redundant nucleotide sequences), Swiss-Prot (A manually annotated and reviewed protein sequence database), KOG (Eukaryotic Ortholog Groups), and GO (Gene Ontology; Mudado Mde and Ortega, 2006). The open reading frame (ORF; Doerks et al., 2002) of each gene was logically generated after successfully compared. Otherwise, Expressed Sequence Tags (EST; Pashley et al., 2006) were used for coding sequence (CDS) prediction to acquire the amino acid sequence of the ORF. Approximately 64.32% of the 43,243 genes possessed their own CDS (27,813). The lengths of all CDS covered a range of 42–7146 bp. The size distributions among Contigs, Unigenes and CDS are given in Figure 2. These results demonstrated that, even though this non-model plant has not been energetically annotated, we still might quickly and economically perform the transcriptome analysis via a NGS approach.

### Functional annotation

**Figure 3:**
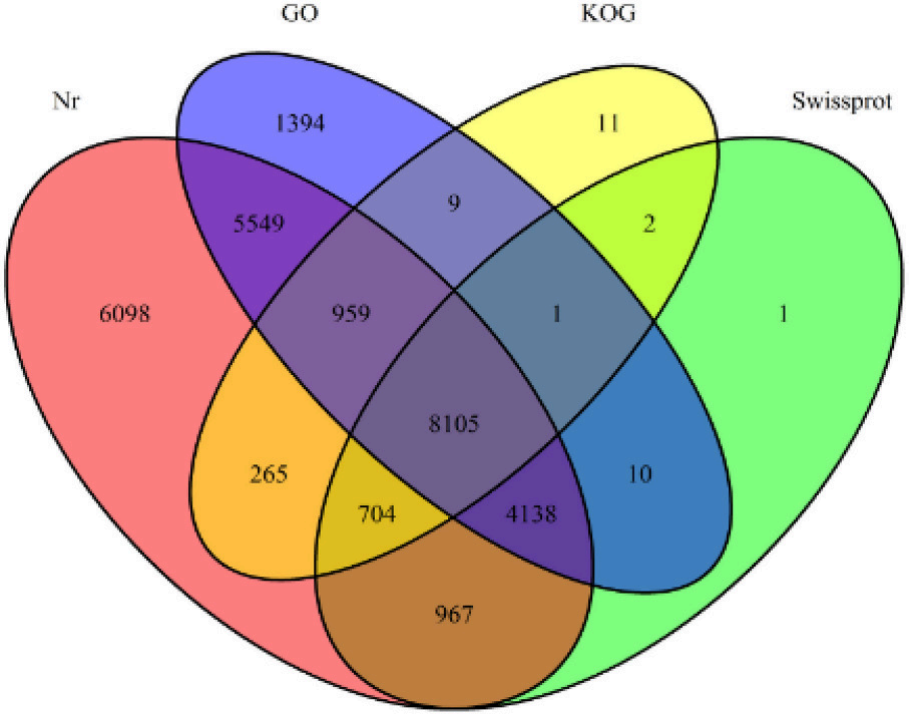
**The number of unigenes annotated by BLASTX with an *E*-value threshold of 10^−−3^ against protein databases**. The numbers in the circles indicate the number of unigenes annotated by single or multiple databases.

Annotation percentages of *D. asperoides* unigenes after being compared with the public databases are summarized in Table 2. The overlapping parts and exclusive sections of 43,243 integrity unigenes among the four databases (Nr, Swiss-Prot, GO, and KOG) are shown in Figure 3. There were 6098, 1, 1394, and 11 unigenes annotated exclusively in these four databases, respectively. 3578 unigenes were annotated in every of the seven public databases. When comparing all unigenes to those from earlier studied species (*Vitis vinifera, Ricinus communis, Populus trichocarpa* and *Glycine max*), 12,304 (28.45%), 3203 (7.41%), 3550 (8.21%), and 1797 (4.16%) annotated unigenes with high similarity were obtained, respectively. Thus, the *D. asperoides* genome was closer to *V. vinifera* than the other species from earlier studies.

**Table 2:** **Statistics of the annotation percentage of *D. asperoides* unigenes compared to public databases**.

**Database**	**Number of unigenes**	**Annotation (%)**
NR	27,546	63.70
NT	11,091	25.64
KO	8781	20.30
Swiss-Prot	19,465	45.01
Pfam	17,490	40.44
GO	20,165	46.63
KOG	10,056	23.25
All annotated unigenes	3,578	8.27
Total unigenes	43,243	100.00

Notably, we also found a positive correlation between the length of unigenes and the hit percentage to sequences from the Nr and Swiss-Prot databases–longer unigenes possessed a greater proportion of hits (Supplementary File S4). In the Nr database, unigenes >1000 bp had higher *E*-values (1e–100) than those <500 bp (1e–50), showing a higher hit percentage: 20,257 (73.54%) unigenes possessed identity of ≥60% with proteins. In the Swiss-Prot database, 12,930(66.43%) unigenes had identity ≥50%. *E*-values (Lolkema and Slotboom, 2005) represent the possibility that the similarity between the other sequences and the target sequence is larger than that of the sequence in random case. So the lower the score, the better the result.

### Gene ontology (GO) classification

**Figure 4:**
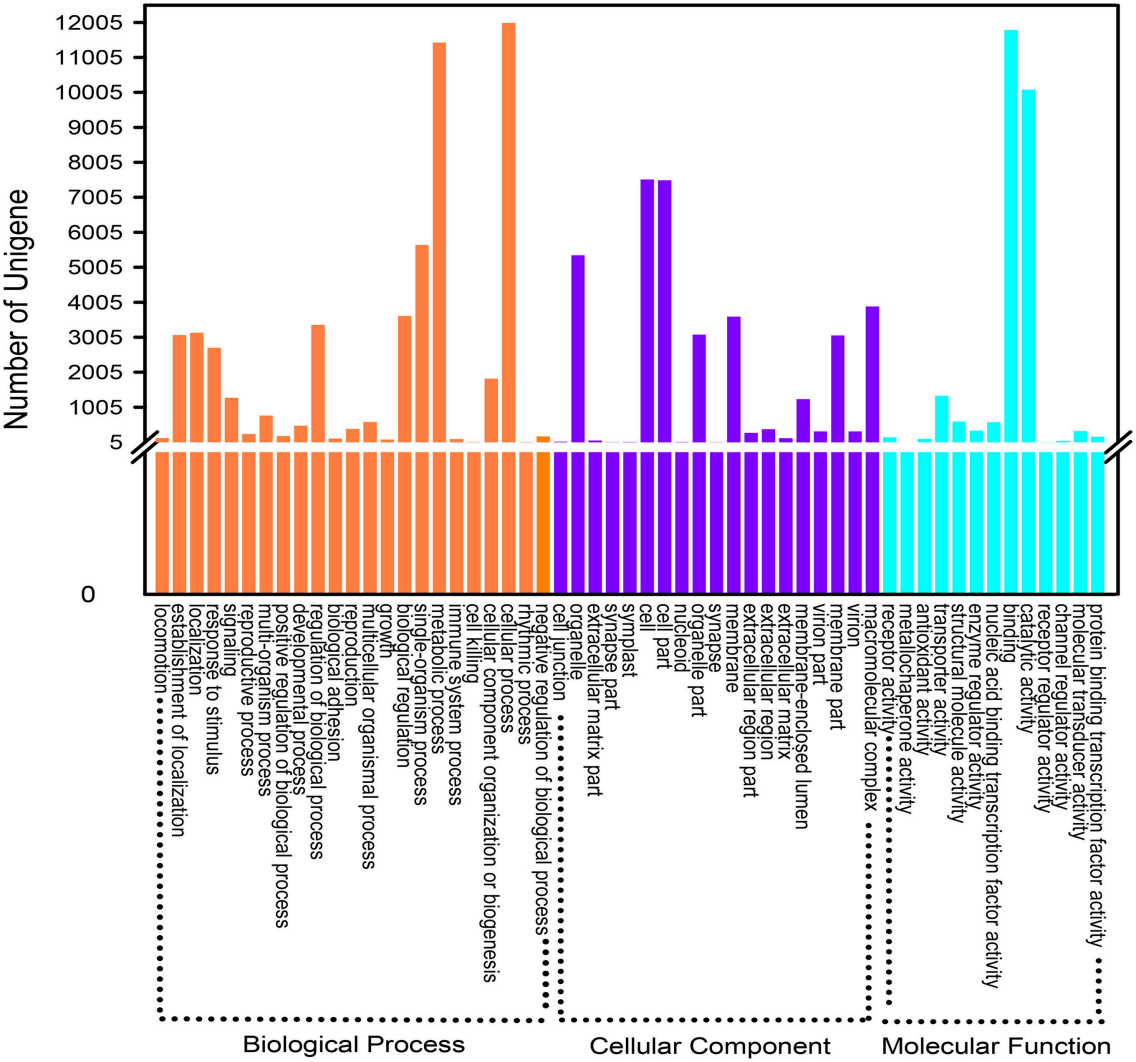
**Gene Ontology classification of assembled unigenes. A total of 20,165 unigenes were categorized into three main categories: “biological process,” “cellular component,” and “molecular function”**.

GO is a classification system to comprehensive descript genes and their products. So far, three independent ontology databases have been built up: biological process (BP), molecular function (MF) and cell component (CC) in GO database. The three ontologies below still possess independently different sub levels, down to form a ontology tree structure. Based on the results of Nr and the Pfam protein database annotations, 20,145 unigenes were successfully hit in one or more ontologies. The detailed descriptions were that 51,266 unigenes were packaged into BP, 36,716 into CC and 25,491 into MF (Figure 4). In a total of 55 ontologies in the three categories, the GO classification distributed all the unigenes to their own functional categories with a high degree of identity. Cellular process (11,987, 47.02%) was the largest group belonging to BP. Most unigenes in CC and MF were allocated to the cell (7517, 29.49%) and the binding (11,787, 46.24%) ontologies, respectively. Without being limited to the above mentioned three categories, all these gene functional annotations will contribute to further exploration of relevant biological genetic information of *D. asperoides*.

### Functional classification by KOG

**Figure 5:**
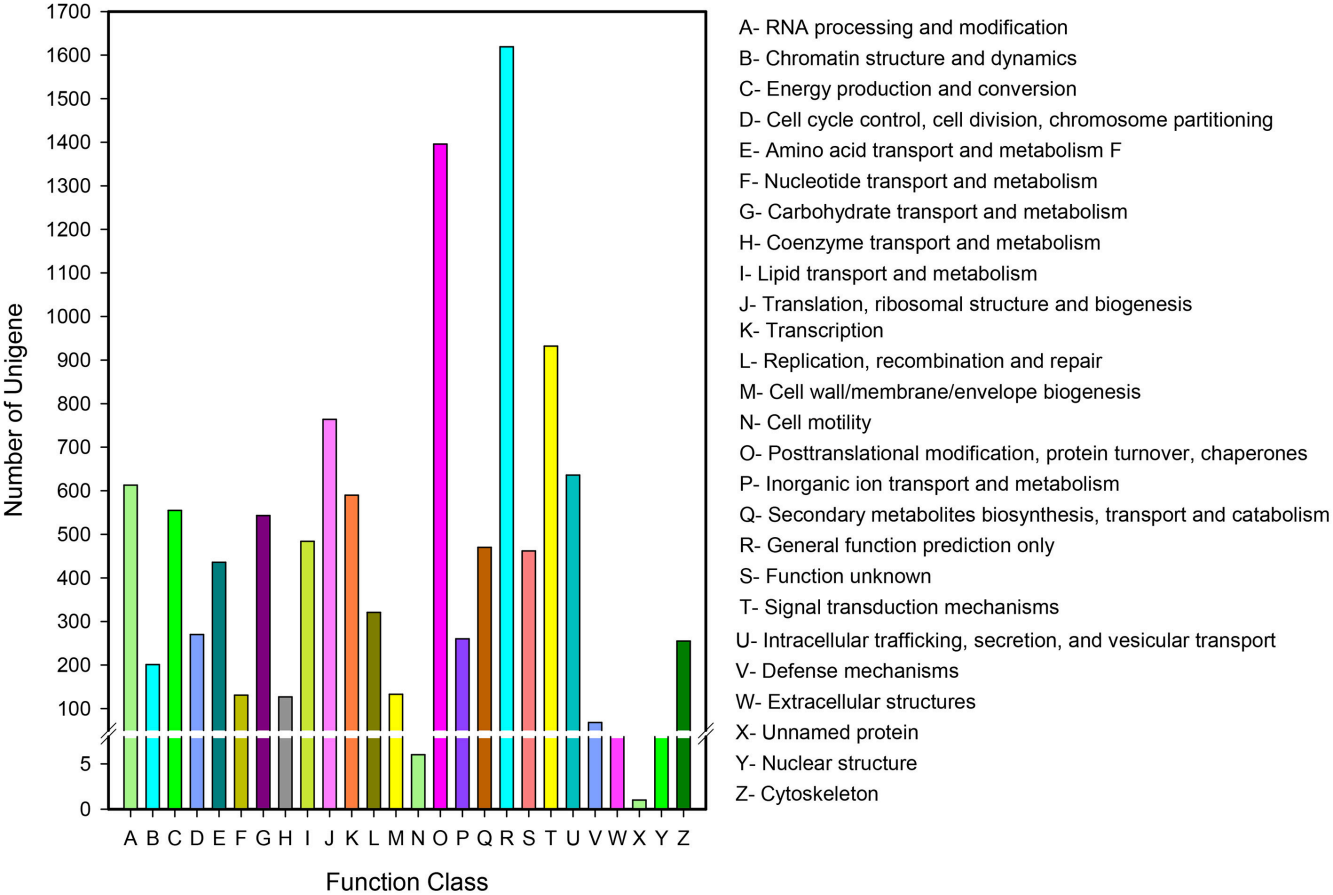
**KOG function classification of *D. asperoides***. All the unigenes were aligned to the KOG database to predict and classify possible functions. A total of 10,056 unigenes were clustered into functional orthologs.

Eukaryotic Ortholog Groups (KOG) annotation system is based on the lineal homologous relationship of the eukaryotic genes in NCBI. KOG which combine with the evolutionary relationships of homologous genes from different species identify and divide those genes into different orthologs (Horiike et al., 2016) clusters. KOG currently have 4852 orthologs. Different genes hold same function if they belong to the same ortholog cluster, naturally, the other members from the same KOG ortholog can directly inherit the functional annotation. Overall, 10,056 of 43,243 unigenes matched to KOG database were clustered into 26 functional orthologs (Figure 5). The largest ortholog was assigned to the general function prediction only cluster (1629 unigenes, 16.10%), followed by posttranslational modification, protein turnover and chaperones (1396, 13.88%), signal transduction mechanisms (932, 9.27%), translation, ribosomal structure, and biogenesis (764, 7.60%).

### KEGG classification

Kyoto Encyclopedia of Genes and Genomes (KEGG) can systematic analyze for the metabolic pathways of gene products and compounds in cells as well as the functions of these gene products. After being subjected to KO annotations, genes could be sorted according to their participation in the KEGG metabolic pathways, and therefore, the function of genome or transcription of newly sequenced species can be definitely clarified. KEGG pathway database is a cross network diagram collecting various metabolic pathways. The transcripts acquired in this study were aligned with the KEGG database and classified into five branches: Cellular Processes (A), Environmental Information Processing (B), Genetic Information Processing (C), Metabolism (D), and Organismal Systems (E). Each branch can be divided into a number of pathways. After we compared all of the unigenes to the KEGG database utilizing BLASTX (E-value threshold was1e–5), 8781 unigenes were found to be of remarkable similarity to proteins from KEGG, all of which could be further mapped to 262 KEGG pathways (Supplementary File S5). The major pathway containing 906 (10.31%) unigenes was translation attached to C (Supplementary File S6). Interestingly, the largest branch was not C but D (4376, 50.12%). Moreover, other highly represented pathways were, carbohydrate metabolism (829, 9.44%), signal transduction (774, 8.81%), folding, sorting, and degradation (731, 8.32%).

**Figure 6:**
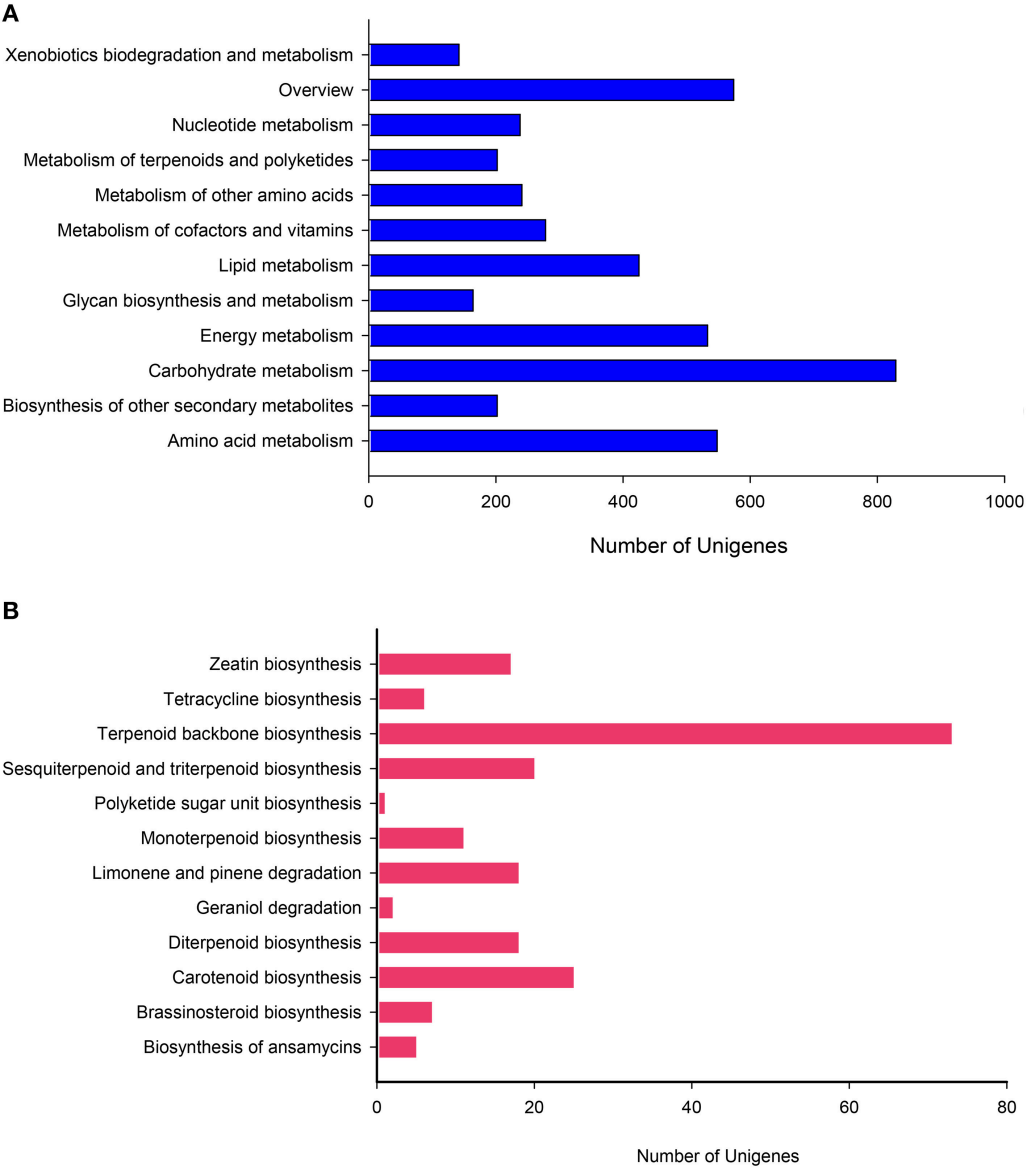
**KEGG pathway mapping for *D. asperoides***. Classification based on **(A)** metabolism branch and **(B)** metabolism of terpenoids and polyketides.

To more thoroughly explore the genes involved in metabolic pathways, the unigenes were mapped to the KEGG metabolism pathway (D). As described above, 4376 genes participated in a total of 130 metabolism pathways of the KEGG database (*E*-value ≤ 1e–5). Among these genes, carbohydrate metabolism (829, 18.94%) was ranked as the first sub-branch in terms of gene numbers (Figure 6A), followed by overview (574, 13.12%) and amino acid metabolism (548, 12.52%), energy metabolism (533, 12.18%), metabolism of other amino acids (241, 5.51%) (Supplementary File S7). Taking into account asperosaponin VI is a kind of triterpenoids from the secondary metabolites, we found, the other secondary metabolites mapped to 15 pathways and metabolism of terpenoids and polyketides discriminatively assigned to 12 pathways covered the equally abundant unigenes (202, 4.62%). Undoubtedly, in the two pathways, those unigenes encoding special proteins may be related to the synthesis of Dipsacus saponin VI.

To further explore the clues, we conducted in-depth analysis to two sub-branches, the biosynthesis of other secondary metabolites and metabolism of terpenoids and polyketides. In the former, Phenylpropanoid biosynthesis pathway (ID: ko00940) containing 128 genes was the dominant group, followed by flavonoid biosynthesis (24, ID: ko00941), tropane, piperidine, and pyridine alkaloid biosynthesis (23, ID: ko00960), stilbenoid, diaryl heptanoid, and gingerol biosynthesis (21, ID: ko00945). In these comparison results, the relevant information about those putative unigenes involved in asporasoponin VI synthesis were still not plainly found, we speculated that the reason may be associated with the complexity of asporasoponin VI precursor.

Most specifically in the metabolism of terpenoids and polyketides, we conducted deep search. The most extensive coverage was terpenoid backbone biosynthesis (73, ID: ko00900; Figure 6B). Carotenoid biosynthesis was the second-largest group (25, ID: ko00906). The third was sesquiterpenoid and triterpenoid biosynthesis (20, ID: ko00909), followed by diterpenoid biosynthesis (18, ID: ko00904), limonene and pinene degradation (18, ID: ko00903), monoterpenoid biosynthesis (11, ID: ko00902). Obviously, clusters 1st and 3rd played major roles in biosynthesis of the main bioactive triterpenoid compounds in *D. asperoides*. This result will be helpful to identify and clarify the function of related enzymes encoded by those genes in the synthesis pathway of asporasoponin VI.

### Candidate genes involved in triterpenoid synthesis

Because triterpenoid saponins are the main active medicinal ingredients of *D. asperoides*, we expanded deep-mining to locate clearly and definitely related candidate genes along the chain of saponin synthesis. Combining the earlier reports on triterpenoid saponin profiles with our study on Illumina sequence analysis of *D. asperoides*, all unigenes encoding enzymes involved in this kind of triterpenoid biosynthesis were revealed.

Primarily, all genes were compared to NCBI database using BLAST searches, and the alignment results are illustrated in Table 3. Specifically, the one or more definite unigenes were successfully matched to known genes encoding functional enzymes that participate in triterpenoid saponin biosynthesis, including AACT (acetyl-CoA acetyltransferase, EC: 2.3.1.9, one unigene), HMGS (hydroxymethylglutaryl-CoA synthase, EC: 2.3.3.10, one unigene), HMGR (hydroxymethylglutaryl-CoA reductase, EC: 1.1.1.34, one unigene), MVK (mevalonate kinase, EC: 2.7.1.36, four unigenes), PMK (phosphomevalonate kinase, EC: 2.7.4.2, two unigenes), MVD (mevalonate diphosphate decarboxylase, EC: 4.1.1.33, one unigene), GPPS (geranylgeranyl pyrophosphate synthase, EC: 2.5.1.29, one unigene), FPPS (farnesyl diphosphate synthase, EC: 2.5.1.10, two unigenes), IPPI (isopentenyl diphosphate isomerase, EC: 5.3.3.2, three unigenes), SS (squalene synthase, EC: 2.5.1.21, five unigenes), SE (squalene epoxidase, EC: 1.14.99.7, three unigenes), β-AS (β-amyrin synthase, EC: 5.4.99.39, six unigenes), and β-A28O (β-amyrin 28-monooxigenase, EC: 1.14.13, one unigene).

**Table 3:** **Unigenes involved in triterpene saponin biosynthesis pathway from *D. asperoides***.

**Gene name**	**EC number**	**Unigene numbers**
AACT, acetyl-CoA acetyltransferase	2.3.1.9	1
HMGS, hydroxymethylglutaryl-CoA synthase	2.3.3.10	1
HMGR, hydroxymethylglutaryl-CoA reductase	1.1.1.34	1
MVK, mevalonate kinase	2.7.1.36	4
PMK, phosphomevalonate kinase	2.7.4.2	2
MVD, mevalonate diphosphate decarboxylase	4.1.1.33	1
GPPS, geranylgeranyl pyrophosphate synthase	2.5.1.29	1
FPPS, farnesyl diphosphate synthase	2.5.1.10	2
IPPI, isopentenyl diphosphate isomerase	5.3.3.2	3
SS, squalene synthase	2.5.1.21	5
SE, squalene epoxidase	1.14.99.7	3
β-AS, β-amyrin synthase	5.4.99.39	6
β-A28O, β-amyrin 28-monooxigenase	1.14.13	1

In addition, even though a one-step reaction catalyzing the synthesis from β-amyrin to oleanolic acid by β-amyrin 28-monooxigenase was initially reported by previous studies, it was revealed that the said reaction is not just one step (Pollier and Goossens, 2012) after all. As oleanolic acid is the precursor for synthesis of the triterpenoid saponins of *D. asperoides*, it is necessary to refine this reaction so that the oleanolic acid profile can be utilized more quickly and easily to produce beneficial drugs for human beings. Therefore, we carried out the related comprehensive survey (Huang et al., 2012; Pollier and Goossens, 2012) and successfully aligned the transcriptome dataset of *D. asperoides* with the four reported reference genes involved in oleanolic acid biosynthesis in NCBI using BLAST (Supplementary File S8). These reference genes (CrCYP716AL1, MtCYP716A12, VvCYP716A17, and VvCYP716A15) share the unique EC number of 1.14.13.cn and each of them can all form one of the same three enzymes of β-amyrin 28-monooxygenase (β-A28O), erythrodiol dehydrogenase (ED), and oleanolic aldehyde 28-monooxygenase (OA28-O; Fukushima et al., 2011). Six candidate genes had high identities of ≥46% with the four reference genes, so they phylogenetically close to the CYP716 family. Scilicet, formation of oleanolic acid can be accomplished with a three-step reaction on the basis of β-amyrin catalyzed by β-A28O, ED, OA28-O. In this process, β-A28O catalyzes the conversion of β-amyrin to erythrodiol, then oleanolic aldehyde and oleanolate can be yielded via catalysis of ED and OA28-O, respectively. The above statements exhibited the vertical pathway associated with the formation and conversion of the oleanolic acid.

**Figure 7:**
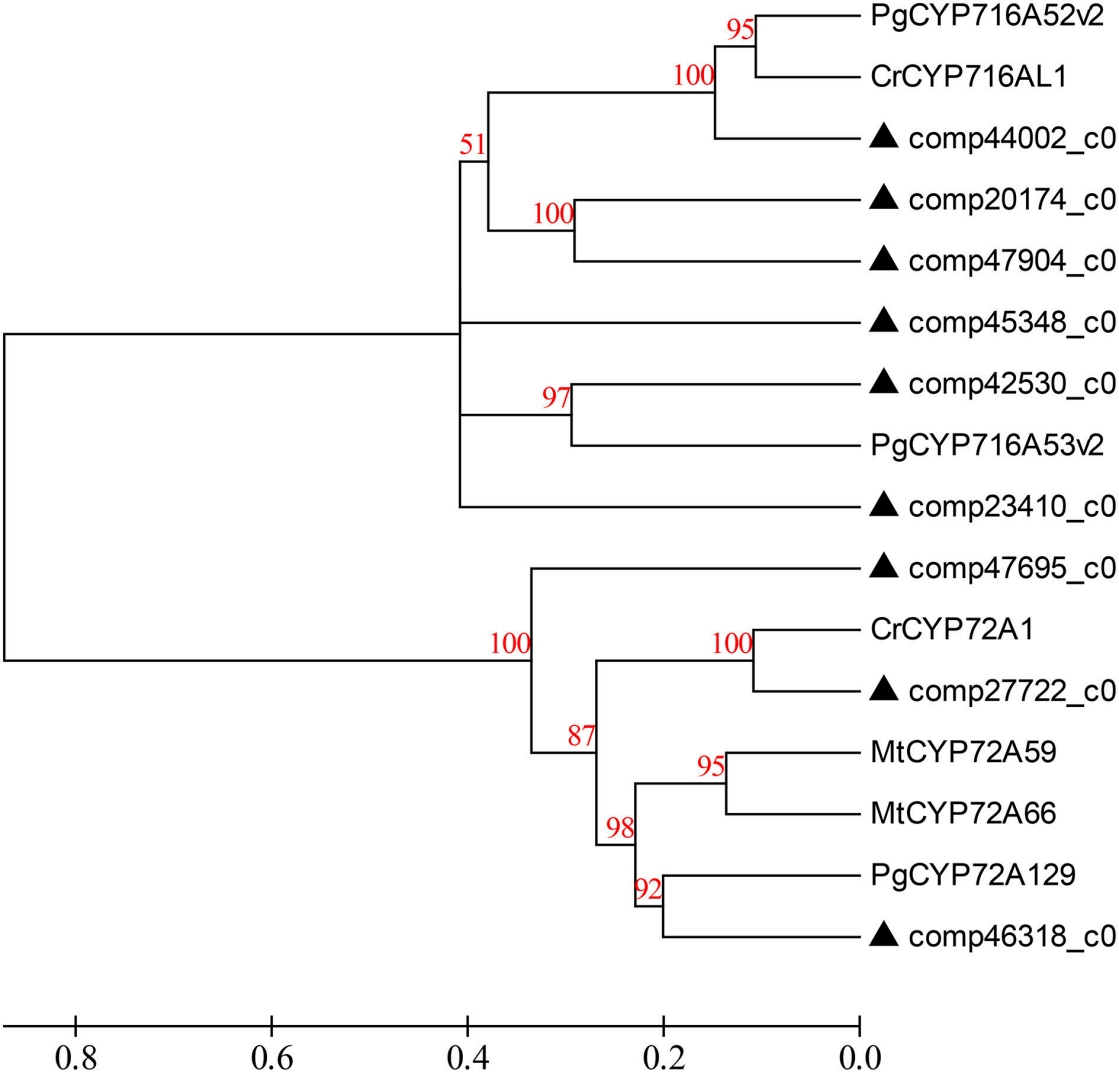
**Phylogenetic tree of the *D. asperoides* CYPs**. Phylogenetic tree constructed based on the deduced amino acid sequences for *D. asperoides* CYPs (filled triangles) and other plant CYPs. Protein sequences were retrieved from NCBI GenBank using the following accession numbers (source organism and proposed function, if any, are given in parentheses): PgCYP716A52v2, AFO63032 (*Panax ginseng*, β-amyrin 28-oxidase); CrCYP716AL1, AEX07773 (*Catharanthus roseus*, in the biosynthesis of oleanolic acid); PgCYP716A53v2, JX036031 (*P. ginseng*, protopanaxadiol 6-hydroxylase); CrCYP72A1, AAA33106 (*C. roseus*, secologanin synthase); MtCYP72A59, ABC59078 (*Medicago truncatula*, cytochrome P450 monooxygenase); MtCYP72A66, ABC59099 (*M. truncatula*, cytochrome P450 monooxygenase); and PgCYP72A129, AEY75218 (*P. ginseng*).

The primary metabolic genes encoding all the enzymes, both known and unknown, involved in the intermediate metabolites up to hederagenin were matched to the corresponding genes from *D. asperoides*. Those candidate CYP450 genes together with the above six genes were used to construct a phylogenetic relationship with reported CYP450 genes from typical plants (Figure 7). Because nearly all genes from the hederagenin pathway had more than one peer in the transcript dataset, we presumed that *D. asperoides* experienced genome replication incidents during anagenesis. Most of all candidate unigenes exhibited high identity to *Medicago truncatula* or other genuine herbaceous plant genes, with >80% homology at the protein level, thus these genes remained highly conserved during evolution.

Since Dipsacus saponin VI is the primary active ingredient in *D. asperoides*, there must exist glycosylases catalyzing glycosylations (Shi et al., 2011) substituted hydroxyl groups of hederagenin by one of four compounds: glucose from UDP-D-glucose, arabinose from UDP-L-arabinose, rhamonose from UDP-L-rhamonose, and xylose from UDP-D-xylose. Obviously, it is rather hederagenin-type (Sarikahya and Kirmizigül, 2012) substrates than oleanane-type (Zhang et al., 2015) triterpenoid substrates which are bonded hydroxyl groups on the kind of carbon skeleton precursors (Liu et al., 2011). We screened the RNA-Seq dataset, and found new genes participating in glycosylation of hederagenin. These new unigenes encode UDP-glucosyltransferase (18), arabisyltransferase (1), xylosyltransferase (13), and rhamsyltransferase (4) (data not shown). As these genes encoding triterpenoid conversion enzymes have not been previously reported, their bioactive verification will be performed in the near future.

**Figure 8:**
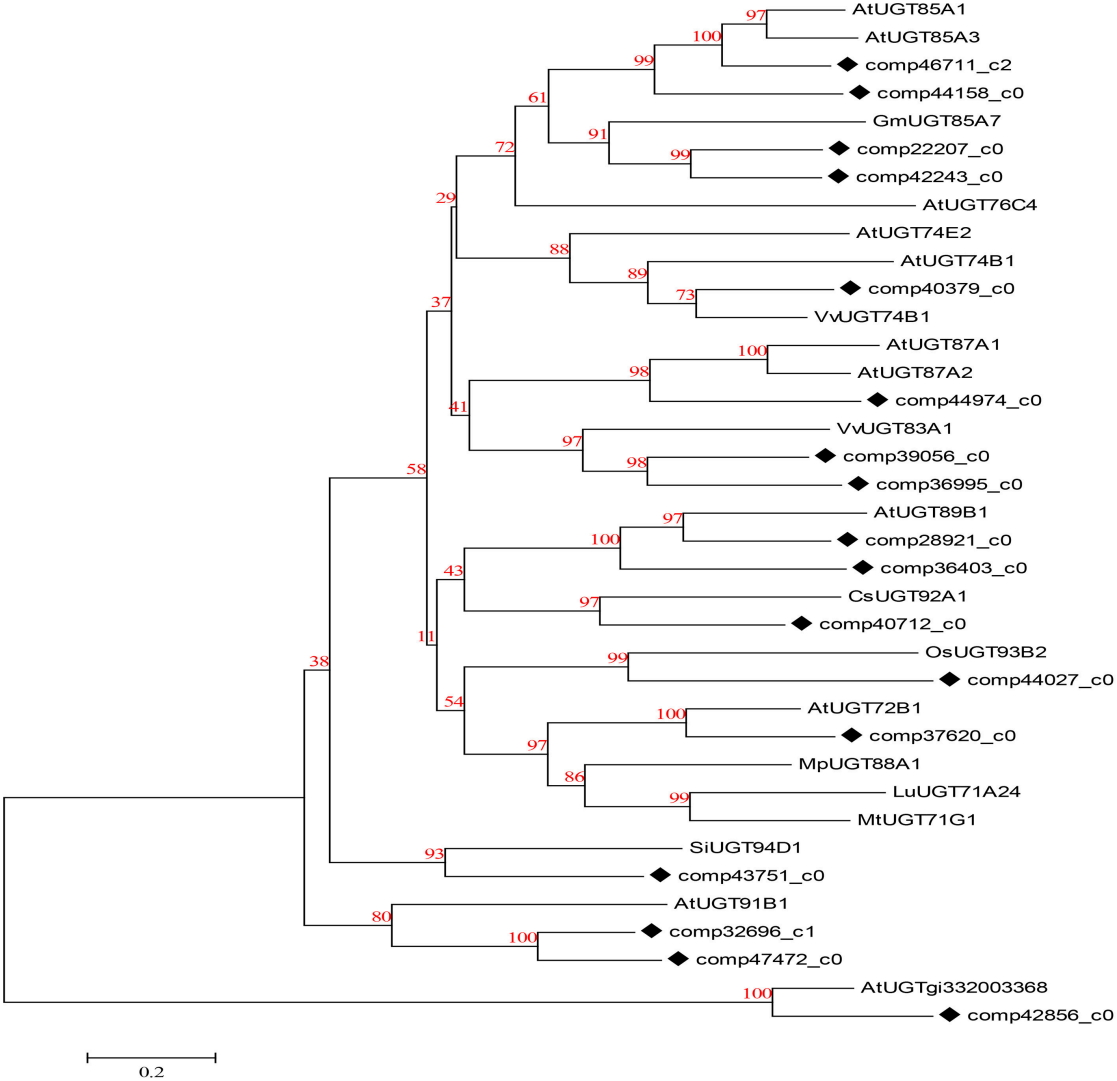
**Phylogenetic tree of *D. asperoides* UGTs**. Phylogenetic tree constructed based on the deduced amino acid sequences for the *D. asperoides* UGTs (solid diamonds) and other plant UGTs. Protein sequences were retrieved from NCBI GeneBank using the following accession numbers (source organisms, if any, are given in parentheses):AtUGT89B1, AEE35520 (*Arabidopsis thaliana*); CsUGT92A1, XP_004142228 (*Cucumis sativus*); OsUGT93B2, CAD41646 (*Oryza sativa*); AtUGT72B1, NP_001190649.1 (*A. thaliana*); MpUGT88A1, ABY73540.1 (*Malus pumila*); LuUGT71A24, AFJ52909 (*Linum usitatissimum*); MtUGT71G1, AAW56092 (*Medicago truncatula*); AtUGT85A1, AEE30237.1 (*A. thaliana*); AtUGT85A3, AEE30236.1 (*A. thaliana*); GmUGT85A7, XP_006577543.1 (*Glycine max*); AtUGT76C4, Q9FI98.1 (*A. thaliana*); AtUGT74E2, AEE27876.1 (*A. thaliana*); AtUGT74B1, AEE30478.1 (*A. thaliana*); VvUGT74B1, XP_002267665.1 (*Vitis vinifera*); AtUGT87A1, AAC16957.1 (*A. thaliana*); AtUGT87A2, O64733.1 (*A. thaliana*); VvUGT83A1, XP_002284331.1 (*V. vinifera*); SiUGT94D1, BAF99027.1 (*Sesamum indicum*); AtUGT91B1, AED98070.1 (*A. thaliana*); and AtUGTgi332003368, AED90751.1 (*A. thaliana*).

UDP-glucosyltransferase (UGT) converts specific substrates to a series of triterpenoid saponins and serves as an extensively scattered taxon of natural products with significant biological functions. For ontology analysis, novel unigenes in our transcriptome database encoding well-known UGTs (Saerens et al., 2011) of *D. asperoides* were further obtained from NCBI or Swiss-Prot databases (Figure 8). The alignment results demonstrated a close evolutionary relationship due to the high degree of similarity. The majority of these unigenes had nearly 100% orthology with reference genes, accordingly, we could infer that the candidate unigenes might have similar functions to the reference genes.

It is worth noting that triterpenoid biosynthesis also includes multiple, parallel sub-pathways, which is caused by the fact that C-3 and C-28 of hederagenin can accept different glycones as substrates. Consequently, triterpenoid biosynthesis seems to more resemble a complex grid than a linear route.

### Verification and analysis of differentially expressed putative genes involved in dipsacus saponin VI synthesis

**Figure 9:**
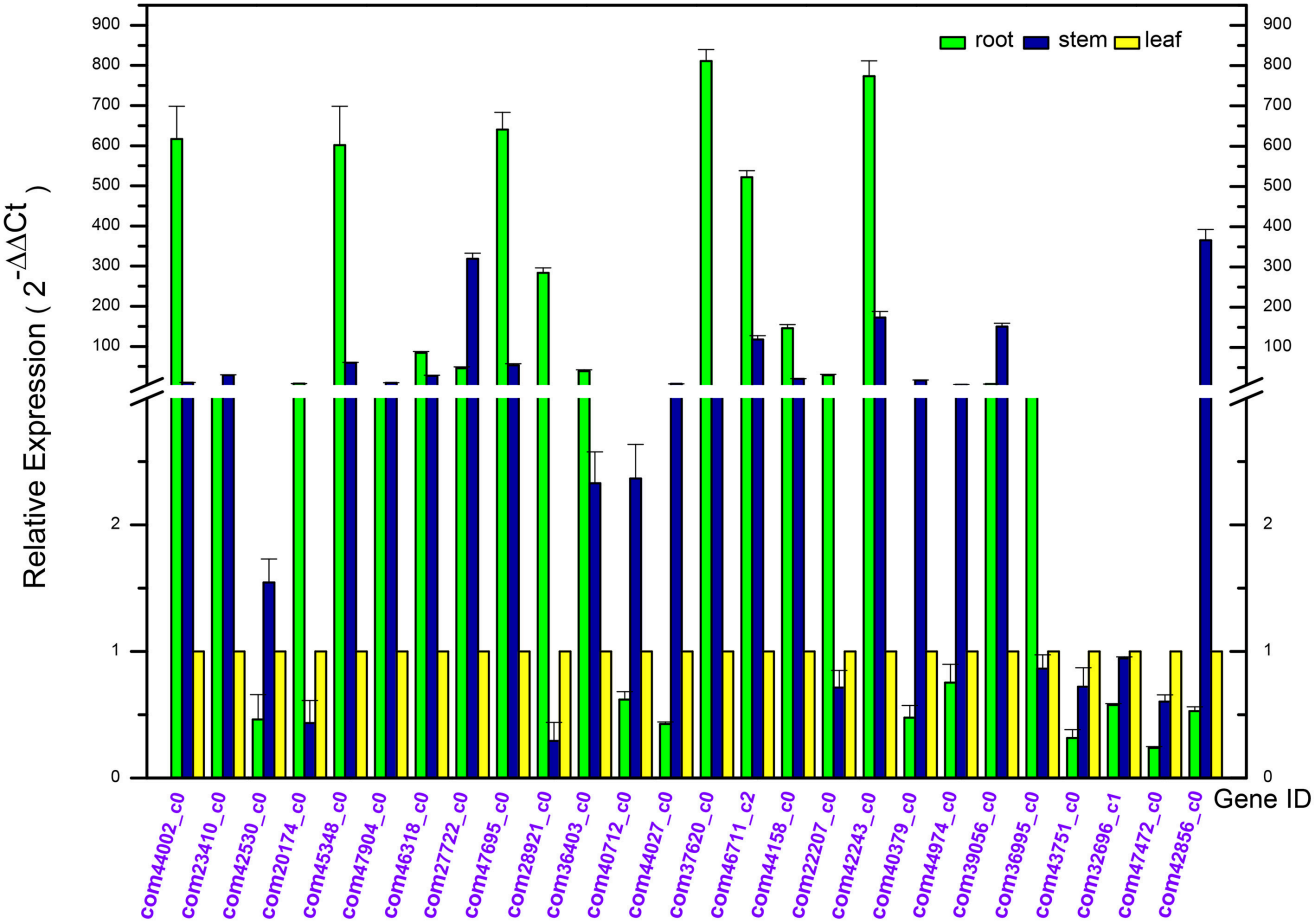
**Differential expression analysis of putative unigenes in the root, stem, and leave of *Dipsacus asperoides* by reverse transcript quantitative PCR (RT-qPCR)**.

In order to confirm the Dipsacus saponin VI related genes actually enrich in the roots, the expression level of 26 related genes in the root, stem and leave of *D. asperoides* were analyzed by reverse transcription quantitative real-time PCR (RT-qPCR; Derveaux et al., 2010; Figure 9). All the PCR reactions with the designed primers produced specific single bands corresponding to each calculated size in agarose gel electrophoresis (data not shown). In this study, we set the gene expression level in leaves of each plant as 1. These gene expression levels in roots or stems were employed to compare with those in leaves from the same plant. The results showed that these genes exhibited different expression levels in the root, stem, and leaves.

Quite a number of these genes were highly expressed in the roots. While the rest of these unigenes exhibited a lower expression level in roots, with highly expressed in stems or leaves, we speculated that might be associated with synthesis of rare saponins. Although the detailed information regarding other special saponins biosynthesis were lacking, not accidentally, the genes were supposed to be abundant in stems and leaves in order to synthesize the special sponins.

In the first nine genes (i.e. deduced CYPs in Dipsacus saponin VI synthesis), five unigenes showed the significantly highest expression level in roots rather than stems and leaves from one plant. Remarkably, the multiples covered a range from several fold to one hundred fold. Likewise, in the last 17 genes (i.e. putative UGTs in Dipsacus saponin VI synthesis), eight genes manifested similar results. Therefore, our results were not unexpected, and indicated that roots may be the main organ for synthesizing Dipsacus saponin VI. Clearly, these enriched genes exhibit higher activities in roots. Despite of the lack of information in Dipsacus saponin VI synthesis currently, we still could deduce that, these enriched unigenes may encode key enzymes for the biosynthesis of Dipsacus saponin VI. The results of qRT-PCR expression analysis will help further study the mechanism of Dipsacus saponin VI biosynthesis.

### Mining and identification of SSR marker

With the development of bioinformatics, SSRs (or microsatellites) have been increasingly utilized in molecular marker screening and genetic breeding studies (Kantartzi et al., 2009). To better understand whether both the genetic diversity and genetic information are closely associated with the biological properties of *D. asperoides*, we sought SSR motifs (Cardle et al., 2000) in all of the unigenes using the MISA search tool. SSR mining in the transcriptome resulted in a total of 4490 SSRs from 43,423 unigenes, SSR analysis results are shown in Table 4. Mono-type motifs were the highest proportion of all motif types (1541, 34.32%), closely followed by tri-(1383, 30.80%), di-(1377, 30.67%), tetra-(151, 3.36%), penta-(24, 0.53%), and hexa-types motif (14, 0.31%). Statistically, 1380 dominant tandems occurred in tri-type motifs with 5–8 times of repeats, followed by 1261 (28.08%) in mono-type with 9–12 times, 1159 (25.81%) in di-type with 5–8 times, and the fewer tandem repeats were not shown. Specifically, 422 (9.40%) T-motif continuing 10 times was the most frequently distributed in the transcriptome. Additionally, the most frequent tri-nucleotide was five GGAs; the most common tetra-nucleotide was nine TTTC units. The fragments of cascaded penta- and hexa- nucleotide types were rarely found, even once (penta-nucleotide repeated 9–12 times). The overall distribution of identified SSRs is shown in Table 5. All of these widely distributed markers will be effective resources for studies at the genetic level and vigorous contribution to genetic diversity in germplasm resources.

**Table 4:** **Statistics of SSR search results in *D. asperoides***.

**Classification**	**Number**
Total number of sequences examined	43,243
Total size of examined sequences (bp)	31,420,741
Total number of identified SSRs	4490
Number of SSR containing sequences	3988
Average number of SSRs per 10 K	1.27
Number of sequences containing more than 1 SSR	456
Number of SSRs present in compound formation	193

**Table 5:** **Distribution of identified SSRs from *D. asperoides* using MISA software**.

**Motif**	**Repeat numbers**					**Total**	**%**
	**5–8**	**9–12**	**13–16**	**17–20**	**21–23**		
Mono-	0	1261	181	47	52	1541	34.32
Di-	1159	218	0	0	0	1377	30.67
Tri-	1380	3	0	0	0	1383	30.80
Tetra-	151	0	0	0	0	151	3.36
Penta-	23	1	0	0	0	24	0.53
Hexa-	10	4	0	0	0	14	0.31
Total	2723	1487	181	47	52	4490	100.00
%	60.65	33.12	4.03	1.05	1.16	100.00	–

## Conclusions

In the first attempt to reveal biological information on *D. asperoides* using the Illumina GAII transcriptome technology, we obtained 43,243 unigenes of high quality, as shown by 97.28% Q20 bases, although without a reference genome sequence. All these unigenes were successfully annotated in public databases. This result presages that mysterious ingredients of many medicinal plants will no longer unfathomable (Rowlands et al., 2015), with the sequencing results of *Salvia miltiorrhiza, Catharanthus roseus*, and other plants (Fulcher and Riha, 2015) being successively reported.

Our study found a large number of candidate genes potentially involved in Dipsacus saponin VI biosynthesis and a total of 3648 SSRs markers. The experimental validation of the putative unigenes exhibited their ample accumulation in roots of *D. asperoides*. These data constitute a new valuable resource for the studies on major metabolism, especially triterpenoid biosynthesis pathway, as well as marker development. These initial bioinformatics will likewise contribute to and promote future research in genetic breeding and synthetic biology (Escobar-Zepeda et al., 2015) on *D. asperoides*.

## Materials and methods

### Ethics statement

No specific approvals were required for the described studies. No special permissions were demanded for the locations and activities. The field studies did not involve endangered or protected species.

### Plant materials and total RNA extraction

*D. asperoides* was cultivated in farmland in Xiangtu township Madden town, Jiangtou village, Dali Bai minority Autonomous Prefecture, Jianchuan County (Zhilei Co. Ltd; East longitude 99°32′05″, north latitude 26°16′11″, altitude 2459 meters). Specimens from five different tissues of the *D. asperoides*, including young roots, tender shoots, young leaves, mature leaves, stems, flower buds, were obtained from ~3-year-old entire plants, thereby ensuring the probability of covering all the genome. The specimens, acquired from local authorities, were promptly frozen in liquid nitrogen and stored at −80°C until RNA extraction.

Total RNA was extracted with RN12-PLANTeasy Reagent Kit (Aidlab Biotechnologies Co. Ltd., Beijing, China) and purified utilizing a RN14-RNAclean Reagent Kit (Aidlab) according to the manufacturer's instructions. The precise concentration of RNA was measured using Qubit® 2.0 Fluorometers (Life Technologies, USA; Simbolo et al., 2013). RNA integrity and quality were assessed using the RNA Nano 6000 Assay Kit of an Agilent Bioanalyzer 2100 (Agilent Technologies, USA) and agarose gel electrophoresis, respectively. Equal amounts of total RNA from each tissue were pooled together for cDNA library preparation.

### cDNA library preparation and sequencing

The poly (A)^+^ RNA was enriched from 20 μg of total RNA pool using magnetic beads cohering Oligo (dT)_25_. Fragmentation buffer was then added to mRNA samples to break mRNA into short segments. The fragmented mRNA samples were used as templates to synthesize the single-strand cDNA with six base random primers (random hexamers). Double-strand cDNA was synthesized by sequentially adding buffer, dNTP, DNA polymerase I, and RNase H, followed by purification using AMPure XP beads. Purified double-strand cDNA was end-repaired, A-tailed. Fragment size selection was performed in AMPure XP beads. Finally, PCR amplification was performed and PCR products were purified in AMPure XP beads to obtain the desired library.

The library was preliminary quantified immediately via Qubit®2.0 Fluorometer (Callaway et al., 2014) and diluted to 1.5 ng/μl, and then the insert size (150–200 bp) of library was detected using the Agilent 2100. If the result was consistent with expectations then the effective concentration (>2 nM) of the library was accurately quantified using Real-time Quantitative PCR. Ultimately, the high quality cDNA libraries were entirely pooled for Illumina paired-end sequencing platform at Novogene Co.Ltd. (Beijing, China). The accession number of the originally high quality reads in the NCBI SRA database is SRA269859.

### Reads dissection and assembly

The reads that either contained adapters, or had the Ns proportion (*N* represents a base of information cannot be determined) of ≥10% of the entire reads, or were low-quality reads (The bases number Qs ≤ 5 accounting for more than 50% of the entire reads.) were all removed using Trinity software (Haas et al., 2013) with the parameter *K*-mer = 25 (Kim et al., 2014). Raw reads were joined together into a clean read. Trinity software was employed to perform *de novo* assemblies of all clean reads. The clean reads containing overlapping areas between them could be assembled into a contig. Via the re-alignment between reads and contigs, different contigs from the same transcript as well as the distance between these contigs could be determined by paired-end reads. Ultimately, the longest unigenes were obtained by connecting and extending the contigs from the same transcript in both ends using paired-end reads. In due course, contigs and unigenes were combined for statistical analysis.

### Function annotation and predicted CDSs

Unigenes were aligned against a full set of public protein databases using BLASTX (Altschul et al., 1997) with *E*-value threshold of 1e–10 to 1e–3: the Nr (NCBI non-redundant), Nt (NCBI nucleotide sequences), Swiss-Prot (Pruitt et al., 2005), Pfam (Protein family; Finn et al., 2008), Kyoto Encyclopedia of Genes and Genomes (KEGG; Kanehisa et al., 2004, 2006), and gene ontology (GO) databases (Harris et al., 2004). The software and methods of functional annotation used for alignment of unigenes to each database were as follows: NCBI blast 2.2.28+ in Nr, Nt, Swiss-Prot, and KOG; HMMER 3.0 package in Pfam; KEGG Automatic Annotation Server in KEGG; and Blast2 GO v2.5 and the writing script in GO functional annotation according to molecular function, biological process and cellular component ontologies. By comparing with the KEGG (Kanehisa et al., 2004) database, we acquired the biological functions and the pathway annotations of the genes.

To further predict and rank possible functions, we attentively analyzed the result of the above comparison. If the comparison was successful, the ORF information box containing the CDS (Hsu and Chen, 2012) of this transcript could be extracted from the results, then this conserved domain could be translated into an amino acid sequence (from 5′to 3′) in accordance with the standard codon table; if the unigene was not mapped to the above protein databases, the CDS could be predicted by Estscan (Iseli et al., 1999; 3.0.3 version) software, thereby obtaining the nucleic acid or amino acid sequences of this conserved domain.

### Phylogenetic analysis

In connection with the deduced amino acid sequences of CYPs and UGTs from *D. asperoides* and other plants, we performed phylogenetic analysis. Clustal X (Selvaraj and Sarma, 2008; using default parameters) was applied to compare all of the above amino acid sequences. Afterwards, phylogenetic distances were analyzed utilizing MEGA5.10 (Tamura et al., 2011) with the parameters set as follows: neighbor-joining in the statistical method, bootstrap method for phylogeny test with 500 replications, uniform rate among sites, complete deletion for gaps/missing data treatment, amino acid for substitution type and the Poisson model. A phylogenetic tree was logically built up. The bootstrap values among the above mentioned amino acid sequences after 500 replications were displayed above each bifurcation. The default distance scale length used was 0.2 amino acid substitutions per site.

### Differentially expression of putative genes involved in dipsacus saponin VI synthesis

Twenty-six genes with assumed activity in Dipsacus saponin VI synthesis were selected for verification using reverse transcription qPCR. All specific primers of these unigenes used for the RT-qPCR assay were designed with primer premier software (Qin et al., 2009; version 6.0) and listed in Supplementary File S9. Total RNA was extracted individually from young roots, stems and young leafs of 1-year-old plant using a modified CTAB method (Zhang et al., 2012) and DNA was removed with RNA purification kit (Tiangen, China). No more than one microgram of total RNA was reverse transcribed to cDNA in total volume of 20 μL in the presence of oligo (dT)_18_ primer according to the protocol of Thermo. The standard curve for each gene was portrayed by real-time PCR with gradient diluted cDNA. The quantitative reaction was performed in 20 μL, containing 10 μL 2 × SYBR Green Master Mix (Toyobo, Japan), 500 nM final concentration of each primer and 1 μL 10-fold diluted cDNA template.

The PCR reactions were run in Roche LightCycler®;96 System using the following program: 95°C for 60 s and 40 cycles of 95°C for 15 s, annealing and extension at 60°C for 60 s. Immediately, the specificity of the individual PCR amplification was validated with Melt curve protocol after the PCR reaction and agarose gel electrophoresis. Three technical replications per three different organs respectively from different plants were performed. Expression levels of four reference genes (actin, GAPDH, 18S, and β-tubulin) in the three organs were analyzed to the degree of conservatism and actin gene was logically granted as an internal control for normalization. Quantifying the relative expression of the genes in three different organs was calculated using the delta-delta Ct method as described by Livak and Schmittgen (Livak and Schmittgen, 2001). All data were represented as the mean ± SD after normalization.

### Mining and identification of SSR marker

Utilizing MIcroSatellite (MISA, http://pgrc.ipk-gatersleben.de/misa/; Thiel et al., 2003) software, the cryptic SSRs containing motifs ranging from mono- to hexa- nucleotides in size were mined from the 43,243 examined sequences. The minimum of contiguous repeat units were arranged as follows: nine for mono-nucleotides and five for di-, tri-, tetra-, penta-, and hexa-nucleotides. After determining the SSR markers, Primer3 (Untergasser et al., 2012) software (2.3.5 version, the default parameters) was utilized to design primer-pairs flanking each SSR.

## Author contributions

Conceived this research: SY, JW. Analyzed the data: GZ, JW, ZG. Contributed plant materials: YL, MH, JC, QH. Wrote the paper: JW, YL. All authors approved the manuscript.

### Conflict of interest statement

The authors declare that the research was conducted in the absence of any commercial or financial relationships that could be construed as a potential conflict of interest.

## Supplementary material

Supplementary File S1 **The underground part of *D. asperoides***.

Supplementary File S2 **The aboveground part of *D. asperoides***.

Supplementary File S3 **Chemical structures of triterpenoid saponins from the root of *D. asperoides***.

Supplementary File S4 **Annotation summary in Nr and Swiss-Prot**.

Supplementary File S5 **KEGG classification count**.

Supplementary File S6 **KEGG classification**.

Supplementary File S7 **KEGG classification**.

Supplementary File S8 **Candidate genes in oleanolic acid synthesis**.

Supplementary File S9 **Specific primers of the candidate unigenes designed for qRT-PCR**.

## References

[B1] Altschul S. F. Madden T. L. Schäffer A. A. Zhang J. Zhang Z. Lipman D. J.. (1997). Gapped BLAST and PSI-BLAST: a new generation of protein database search programs. Nucleic Acids Res. 25, 3389–3402. 10.1093/nar/25.17.3389 9254694 PMC146917

[B2] Callaway J. L. Huang S. Karampetsou E. Crolla J. A. (2014). Perspective on the technical challenges involved in the implementation of array-CGH in prenatal diagnostic testing. Mol. Biotechnol. 56, 312–318. 10.1007/s12033-013-9710-4 24146428

[B3] Cardle L. Ramsay L. Milbourne D. Macaulay M. Marshall D. Waugh R. (2000). Computational and experimental characterization of physically clustered simple sequence repeats in plants. Genetics 156, 847–854. 11014830 10.1093/genetics/156.2.847 PMC1461288

[B4] Cock P. J. Fields C. J. Goto N. Heuer M. L. Rice P. M. (2010). The Sanger FASTQ file format for sequences with quality scores, and the Solexa/Illumina FASTQ variants. Nucleic Acids Res. 38, 1767–1771. 10.1093/nar/gkp1137 20015970 PMC2847217

[B5] Corcelle E. Nebout M. Bekri S. Gauthier N. Hofman P. Poujeol P.. (2006). Disruption of autophagy at the maturation step by the carcinogen lindane is associated with the sustained mitogen-activated protein kinase/extracellular signal-regulated kinase activity. Cancer Res. 66, 6861–6870. 10.1158/0008-5472.CAN-05-3557 16818664

[B6] Derveaux S. Vandesompele J. Hellemans J. (2010). How to do successful gene expression analysis using real-time PCR. Methods 50, 227–230. 10.1016/j.ymeth.2009.11.001 19969088

[B7] Doerks T. Copley R. R. Schultz J. Ponting C. P. Bork P. (2002). Genome annotation past, present, and future: how to define an ORF at each locus. Genome Res. 12, 47–56. 10.1101/gr.203201 16339376

[B8] Earl D. Bradnam K. St. John J. Darling A. Lin D. Fass J.. (2011). Assemblathon 1: a competitive assessment of *de novo* short read assembly methods. Genome Res. 21, 2224–2241. 10.1101/gr.126599.111 21926179 PMC3227110

[B9] Escobar-Zepeda A. A. Vera-Ponce de León A. Sanchez-Flores A. (2015). The road to metagenomics: from microbiology to DNA sequencing technologies and bioinformatics. Front. Genet. 6:348. 10.3389/fgene.2015.00348 26734060 PMC4681832

[B10] Finn R. D. Tate J. Mistry J. Coggill P. C. Sammut S. J. Bateman A.. (2008). The Pfam protein families database. Nucleic Acids Res. 36 (Database issue), D281–D288. 10.1093/nar/gkv1344 18039703 PMC2238907

[B11] Fu N. Wang Q. Shen H. L. (2013). *De novo* assembly, gene annotation and marker development using Illumina paired-end transcriptome sequences in celery (*Apium graveolens* L.). PLoS ONE 8:e57686. 10.1371/journal.pone.0057686 23469050 PMC3585167

[B12] Fukushima E. O. Seki H. Ohyama K. Ono E. Umemoto N. Mizutani M.. (2011). CYP716A subfamily members are multifunctional oxidases in triterpenoid biosynthesis. Plant Cell Physiol. 52, 2050–2061. 10.1093/pcp/pcr146 22039103

[B13] Fulcher N. Riha K. (2015). Using centromere mediated genome elimination to elucidate the functional redundancy of candidate telomere binding proteins in *Arabidopsis thaliana*. Front. Genet. 6:349. 10.3389/fgene.2015.00349 26779251 PMC4700174

[B14] Grabherr M. G. Haas B. J. Yassour M. Levin J. Z. Thompson D. A. Regev A.. (2011). Full-length transcriptome assembly from RNA-Seq data without a reference genome. Nat. Biotechnol. 29, 644–652. 10.1038/nbt.1883 21572440 PMC3571712

[B15] Haas B. J. Papanicolaou A. Yassour M. Grabherr M. Blood P. D. Regev A.. (2013). *De novo* transcript sequence reconstruction from RNA-seq using the Trinity platform for reference generation and analysis. Nat. Protocols 8, 1494–1512. 10.1038/nprot.2013.084 23845962 PMC3875132

[B16] Harris M. A. Clark J. Ireland A. Lomax J. Ashburner M. Foulger R.. (2004). The Gene Ontology (GO) database and informatics resource. Nucleic Acids Res. 32 (Database issue), D258–D261. 10.1093/nar/gkh036 14681407 PMC308770

[B17] Horiike T. Minai R. Miyata D. Nakamura Y. Tateno Y. (2016). Ortholog-finder: a tool for constructing an ortholog dataset. Genome Biol. Evol. 8, 446–457. 10.1093/gbe/evw005 26782935 PMC4779612

[B18] Hsu M. K. Chen F. C. (2012). Selective constraint on the upstream open reading frames that overlap with coding sequences in animals. PLoS ONE 7:e48413. 10.1371/journal.pone.0048413 23133632 PMC3486843

[B19] Huang L. Li J. Ye H. Li C. Wang H. Liu B.. (2012). Molecular characterization of the pentacyclic triterpenoid biosynthetic pathway in *Catharanthus roseus*. Planta 236, 1571–1581. 10.1007/s00425-012-1712-0 22837051

[B20] Iseli C. Jongeneel C. V. Bucher P. (1999). ESTScan: a program for detecting, evaluating, and reconstructing potential coding regions in EST sequences. Proc. Int. Conf. Intell. Syst. Mol. Biol. 138–148. 10786296

[B21] Kanehisa M. Goto S. Hattori M. Aoki-Kinoshita K. F. Itoh M. Hirakawa M.. (2006). From genomics to chemical genomics: new developments in KEGG. Nucleic Acids Res. 34 (Database issue), D354–D357. 10.1093/nar/gkj102 16381885 PMC1347464

[B22] Kanehisa M. Goto S. Kawashima S. Okuno Y. Hattori M. (2004). The KEGG resource for deciphering the genome. Nucleic Acids Res. 32 (Database issue), D277–D280. 10.1093/nar/gkh063 14681412 PMC308797

[B23] Kantartzi S. K. Ulloa M. Sacks E. Stewart J. M. (2009). Assessing genetic diversity in Gossypium arboreum L. cultivars using genomic and EST-derived microsatellites. Genetica 136, 141–147. 10.1007/s10709-008-9327-x 18853261

[B24] Kim J. H. Roh J. Y. Kwon D. H. Kim Y. H. Yoon K. A. Lee S. H.. (2014). Estimation of the genome sizes of the chigger mites *Leptotrombidium pallidum* and *Leptotrombidium scutellare* based on quantitative PCR and k-mer analysis. Parasit. Vectors 7:279. 10.1186/1756-3305-7-279 24947244 PMC4079623

[B25] Liu J. J. Wang X. L. Guo B. L. Huang W. H. Xiao P. G. Tu G. Z.. (2011). Triterpenoid saponins from Dipsacus asper and their activities *in vitro*. J. Asian Nat. Prod. Res. 13, 851–860. 10.1080/10286020.2011.598858 21830891

[B26] Liu Y. Wei Y. F. Zhu J. P. Long F. Guo S. S. Wang H. D.. (2010). Fingerprint analysis of *Dipsacus asperoides* by HPLC. Zhong Yao Cai 33, 359–361. 20681298

[B27] Livak K. J. Schmittgen T. D. (2001). Analysis of relative gene expression data using real-time quantitative PCR and the 2(-Delta Delta C(T)) Method. Methods 25, 402–408. 10.1006/meth.2001.1262 11846609

[B28] Lolkema J. S. Slotboom D. J. (2005). Sequence and hydropathy profile analysis of two classes of secondary transporters. Mol. Membr. Biol. 22, 177–189. 10.1080/09687860500063324 16096261

[B29] Moreton J. Izquierdo A. Emes R. D. (2015). Assembly, assessment, and availability of *de novo* generated eukaryotic transcriptomes. Front. Genet. 6:361. 10.3389/fgene.2015.00361 26793234 PMC4707302

[B30] Mudado Mde A. Ortega J. M. (2006). A picture of gene sampling/expression in model organisms using ESTs and KOG proteins. Genet. Mol. Res. 5, 242–253. 16755515

[B31] Niu Y. Li C. Pan Y. Li Y. Kong X. Wang S.. (2015). Treatment of Radix Dipsaci extract prevents long bone loss induced by modeled microgravity in hindlimb unloading rats. Pharm. Biol. 53, 110–116. 10.3109/13880209.2014.911920 25243871

[B32] Pashley C. H. Ellis J. R. McCauley D. E. Burke J. M. (2006). EST databases as a source for molecular markers: lessons from Helianthus. J. Hered. 97, 381–388. 10.1093/jhered/esl013 16840524

[B33] Pollier J. Goossens A. (2012). Oleanolic acid. Phytochemistry 77, 10–15. 10.1016/j.phytochem.2011.12.022 22377690

[B34] Pruitt K. D. Tatusova T. Maglott D. R. (2005). NCBI Reference Sequence (RefSeq): a curated non-redundant sequence database of genomes, transcripts and proteins. Nucleic Acids Res. 33 (Database issue), D501–D504. 10.1093/nar/gki025 15608248 PMC539979

[B35] Qin P. Z. Niu C. G. Zeng G. M. Ruan M. Tang L. Gong J. L. (2009). Time-resolved fluorescence based DNA detection using novel europium ternary complex doped silica nanoparticles. Talanta 80, 991–995. 10.1016/j.talanta.2009.08.027 19836584

[B36] Ramsay L. Macaulay M. Ivanissevich S. D. (2000). A simple sequence repeat-based linkage Map of Barley. Genet. Soc. Am. 156, 1997–2005. 11102390 10.1093/genetics/156.4.1997 PMC1461369

[B37] Rowlands H. J. Abbasi S. Yankulov K. Wyse B. A. (2015). Book review: epigenetics: current research and emerging trends. Front. Genet. 6:347 10.3389/fgene.2015.00347

[B38] Saerens K. M. Roelants S. L. Van Bogaert I. N. Soetaert W. (2011). Identification of the UDP-glucosyltransferase gene UGTA1, responsible for the first glucosylation step in the sophorolipid biosynthetic pathway of Candida bombicola ATCC 22214. FEMS Yeast Res. 11, 123–132. 10.1111/j.1567-1364.2010.00695.x 21073653

[B39] Sarikahya N. B. Kirmizigül S. (2012). Antimicrobially active hederagenin glycosides from Cephalaria elmaliensis. Planta Med. 78, 828–833. 10.1055/s-0031-1298415 22495443

[B40] Seifert-Klauss V. Prior J. C. (2010). Progesterone and bone: actions promoting bone health in women. J. Osteoporos. 2010:845180. 10.4061/2010/845180 21052538 PMC2968416

[B41] Selvaraj D. Sarma R. K. (2008). Phylogenetic analysis of chloroplast matK genefrom Zingiberaceae for plant DNA barcoding. Bioinformation 3, 24–27. 19052662 10.6026/97320630003024 PMC2586133

[B42] Seong E. S. Yoo J. H. Choi J. H. Kim C. H. Jeon M. R. Yu C. Y.. (2015). Expressed sequence tags analysis and design of simple sequence repeats markers from a full-length cDNA Library in *Perilla frutescens* (L.). Int. J. Genomics 2015:679548. 10.1155/2015/679548 26664999 PMC4668317

[B43] Shi C. Y. Yang H. Wei C. L. Yu O. Zhang Z. Z. Wan X. C.. (2011). Deep sequencing of the Camellia sinensis transcriptome revealed candidate genes for major metabolic pathways of tea-specific compounds. BMC Genomics 12:131. 10.1186/1471-2164-12-131 21356090 PMC3056800

[B44] Simbolo M. Gottardi M. Corbo V. Fassan M. Mafficini A. Malpeli G.. (2013). DNA qualification workflow for next generation sequencing of histopathological samples. PLoS One 8:e62692. 10.1371/journal.pone.0062692 23762227 PMC3675123

[B45] Somers D. J. Kirkpatrick R. Moniwa M. Walsh A. (2003). Mining single-nucleotide polymorphisms from hexaploid wheat ESTs. Genome 46, 431–437. 10.1139/g03-027 12834059

[B46] Strickler S. R. Bombarely A. Mueller L. A. (2012). Designing a transcriptome next-generation sequencing project for a nonmodel plant species. Am. J. Bot. 99, 257–266. 10.3732/ajb.1100292 22268224

[B47] Suter B. Zhang X. Pesce C. G. Mendelsohn A. R. Dinesh-Kumar S. P. Mao J. H. (2015). Next-generation sequencing for binary protein-protein interactions. Front. Genet. 6:346. 10.3389/fgene.2015.00346 26734059 PMC4681833

[B48] Tamura K. Peterson D. Peterson N. Stecher G. Nei M. Kumar S. (2011). MEGA5: molecular evolutionary genetics analysis using maximum likelihood, evolutionary distance, and maximum parsimony methods. Mol. Biol. Evol. 28, 2731–2739. 10.1093/molbev/msr121 21546353 PMC3203626

[B49] Thiel T. Michalek W. Varshney R. K. Graner A. (2003). Exploiting EST databases for the development and characterization of gene-derived SSR-markers in barley (Hordeum vulgare L.). Theor. Appl. Genet. 106, 411–422. 10.1007/s00122-002-1031-0 12589540

[B50] Untergasser A. Cutcutache I. Koressaar T. Ye J. Faircloth B. C. Rozen S. G.. (2012). Primer3–new capabilities and interfaces. Nucleic Acids Res. 40, e115. 10.1093/nar/gks596 22730293 PMC3424584

[B51] Wong R. W. Rabie A. B. Hägg E. U. (2007). The effect of crude extract from Radix Dipsaci on bone in mice. Phytother. Res. 21, 596–598. 10.1002/ptr.2126 17380551

[B52] Zhang X. Wang L. Shou L. (2012). A rapid modifie CTAB method of extracting genomic DNA from wheat leaf. Chin. Agric. Sci. Bull. 28, 46–49.

[B53] Zhang Y. Kiyohara H. Matsumoto T. Yamada H. (1997). Fractionation and chemical properties of immunomodulating polysaccharides from roots of *Dipsacus asperoides*. Planta Med. 63, 393–399. 10.1055/s-2006-957720 9342940

[B54] Zhang Y. Li X. Ruan J. Wang T. Dong Y. Hao J.. (2015). Oleanane type saponins from the stems of *Astragalus membranaceus* (Fisch.) Bge. *var. mongholicus* (Bge.) Hsiao. Fitoterapia 109, 99–105. 10.1016/j.fitote.2015.12.006 26687558

[B55] Zhang Z. J. Qian Y. H. Hu H. T. Yang J. Yang G. D. (2003). The herbal medicine Dipsacus asper wall extract reduces the cognitive deficits and overexpression of beta-amyloid protein induced by aluminum exposure. Life Sci. 73, 2443–2454. 10.1016/S0024-3205(03)00649-0 12954453

[B56] Zhu X. Bensoussan A. Zhu L. Qian J. Xu M. Zhou C.. (2009). Primary dysmenorrhoea: a comparative study on Australian and Chinese women. Complement. Ther. Med. 17, 155–160. 10.1016/j.ctim.2008.10.001 19398069

