# Supplementary material for: Genome-Wide Transcriptional Excavation of Dipsacus asperoides Unmasked both Cryptic Asperosaponin Biosynthetic Genes and SSR Markers
Source: Front Plant Sci. 2016 Mar 29;7:339. doi: 10.3389/fpls.2016.00339 (PMC4809893; doi:10.3389/fpls.2016.00339)
Supplement: Supplementary File S1 — The underground part of D. asperoides. [file Presentation1.ZIP › suport file/File S3. Chemical structures of triterpenoidsaponins.docx]

**File S3.** **Chemical structures of triterpenoidsaponins from the root of *Dipsacusasperoides.***

| Saponins | Aglycone | R1 | R2 |
| --- | --- | --- | --- |
| HN saponin F | hederagenin | Ara- | Glc- |
| Dipsacussaponin VI |  | Ara- | Glc-glc- |
| Macranthoidin A |  | Glc-rha-ara- | Glc-glc- |
| Dipsacussaponin J |  | Rha-glc(glc)-rha-ara- | Glc- |
| DipsacussaponinXll |  | Rha-glc(glc)-rha-ara- | Glc-glc- |
| DipsacussaponinX |  | Rha-glc(glc-rha)-rha-ara- | Glc-glc- |
| 3-О-β-D-xylopyranosyl(1→4)  -β-D-glucopyranosyl-(1→4)]- [a-L-rhamnopyranosyl(1→3)]  -β-D-glucopyranosyl(1→3)-a-  L-Rhamnopyranosyl-(1→2)-a-  L-arabinopyranosyl-hederagenin  -28-O-β-D-glucopyranoside |  | Rha-glc(glc-rha)-rha-ara- | Glc- |
| Dipsacussaponin K |  | Ara- | Glc-glc-glc- |
