# Supplementary figures and images for: Genome-Wide Transcriptional Excavation of Dipsacus asperoides Unmasked both Cryptic Asperosaponin Biosynthetic Genes and SSR Markers
Source: Front Plant Sci. 2016 Mar 29;7:339. doi: 10.3389/fpls.2016.00339 (PMC4809893; doi:10.3389/fpls.2016.00339)

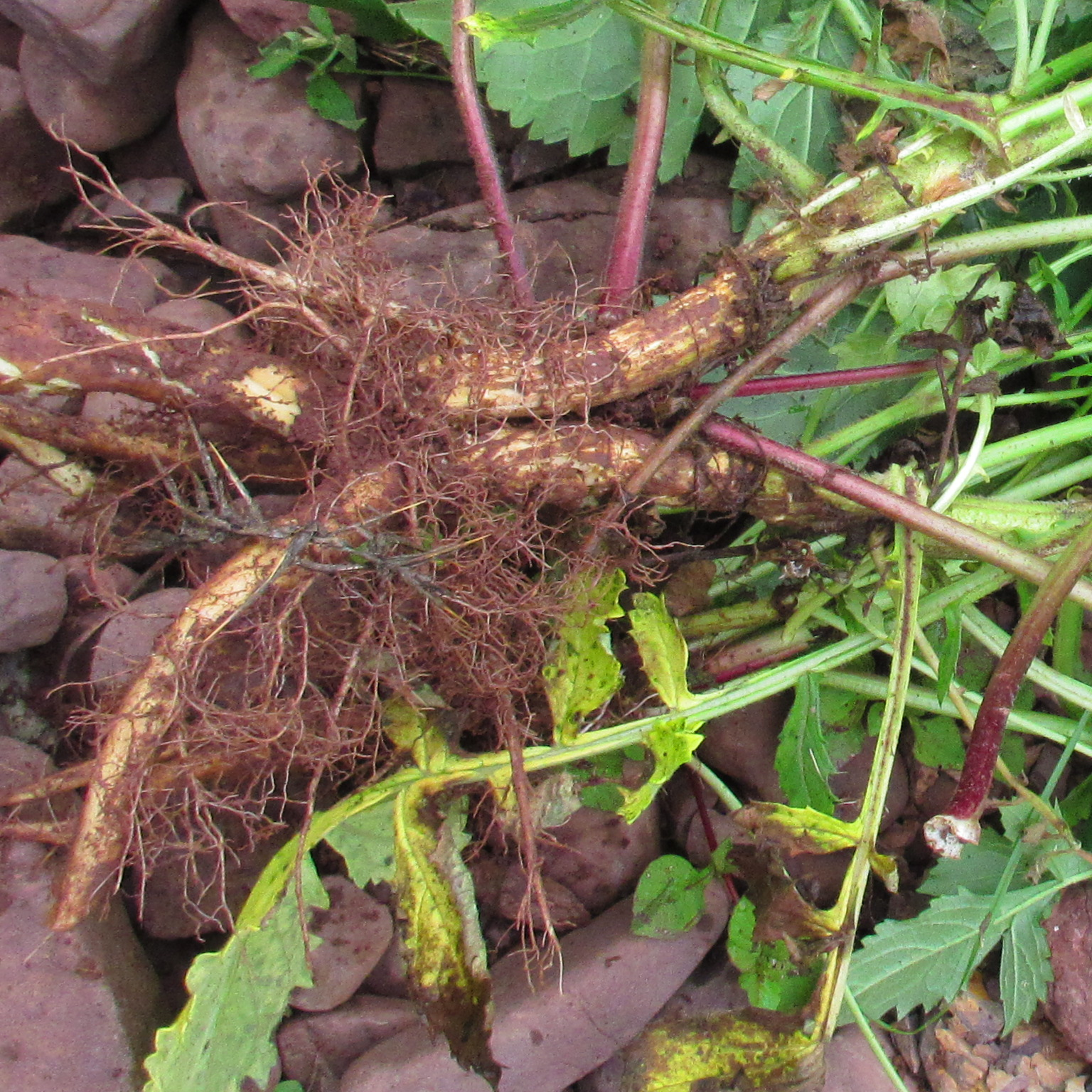

Supplement: Supplementary File S1 — The underground part of D. asperoides. [file Presentation1.ZIP › suport file/File S1. The underground partú¡root of Dipsacus asperoides.tif]

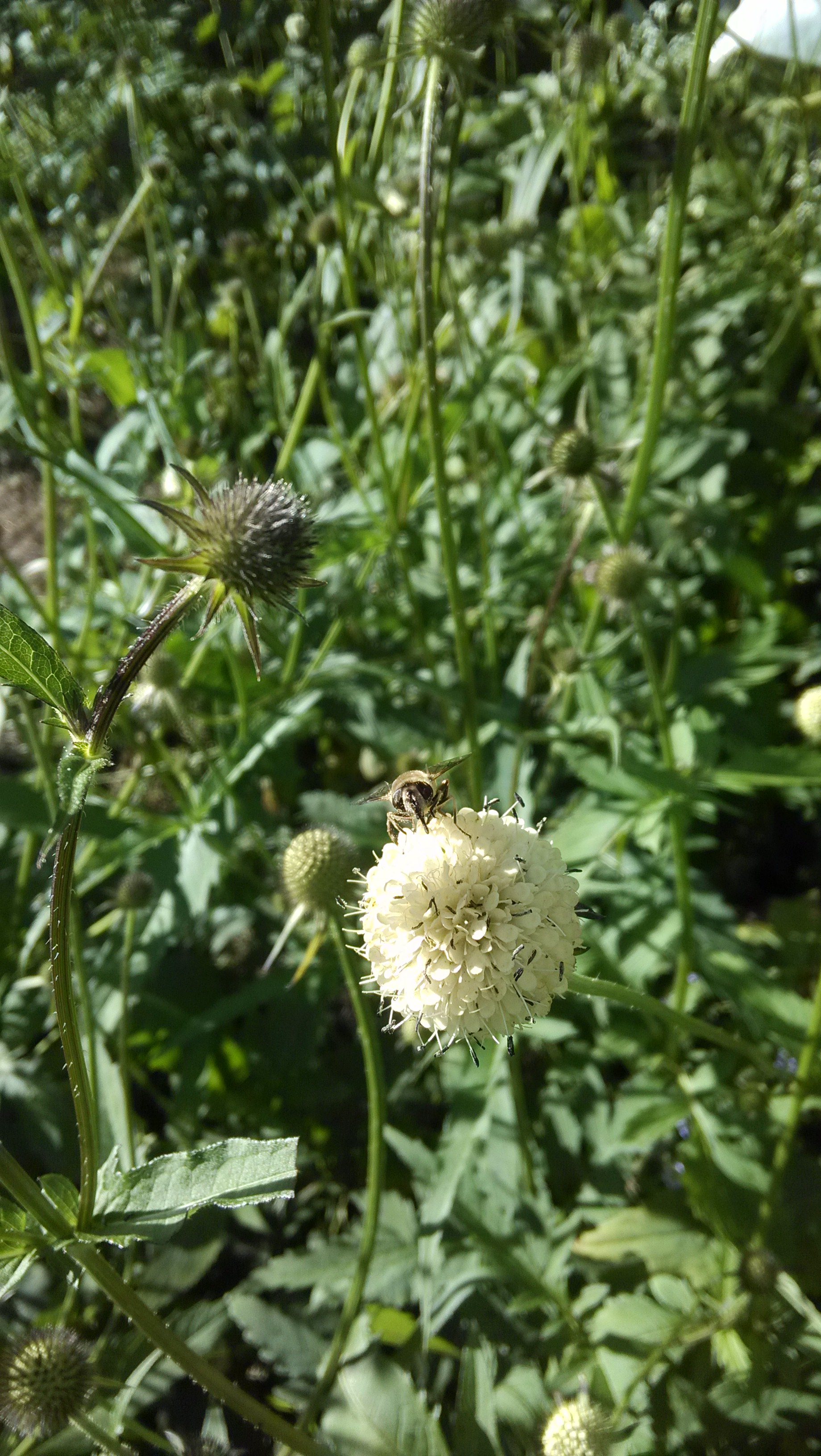

Supplement: Supplementary File S1 — The underground part of D. asperoides. [file Presentation1.ZIP › suport file/File S2. The ground part of Dipsacus asperoides.tif]

KEGG Classification

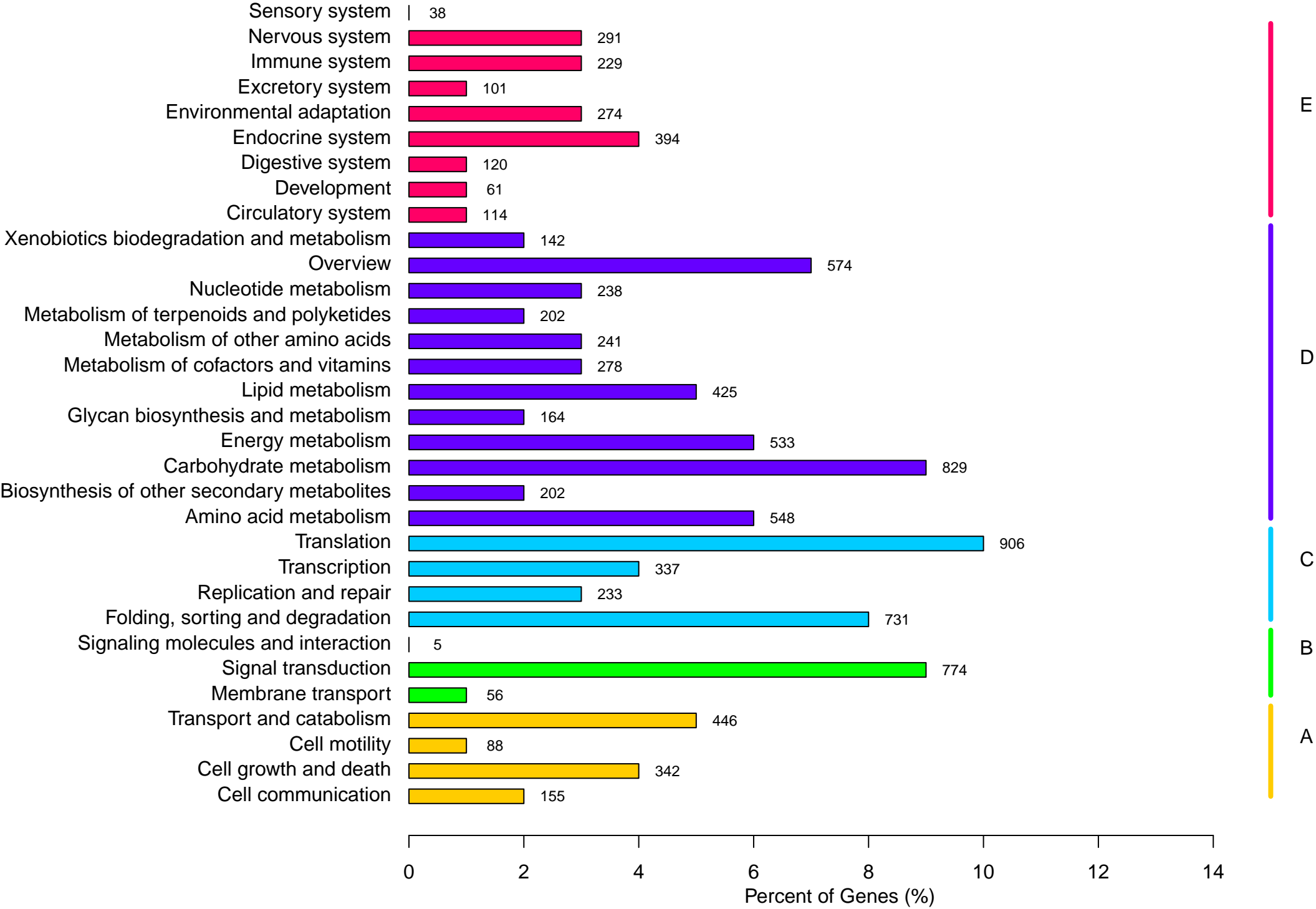

Supplement: Supplementary File S1 — The underground part of D. asperoides. [file Presentation1.ZIP › suport file/File S6. KEGG_classification.pdf]
